# Visual Neural Dynamics of Adolescents with Myopia During Face Inversion: An ERP and Oscillatory Study

**DOI:** 10.3390/brainsci15121312

**Published:** 2025-12-09

**Authors:** Peng Chen, Denise Koh, Yusha Gu, Hongwei Wang, Weiping Du

**Affiliations:** 1 School of Physical Education, Ningxia Normal University, Guyuan 756099, China; p117279@siswa.ukm.edu.my (P.C.); guyusha@stu.nxnu.edu.cn (Y.G.); 2 Center for Sports and Health Research, Ningxia Normal University, Guyuan 756099, China; 3 Faculty of Education, National University of Malaysia, Bangi 43600, Selangor, Malaysia; 4 School of Physical Education, Northwest Normal University, Lanzhou 730070, China

**Keywords:** myopia, event-related potential (ERP), face inversion effect, early visual processing, adolescents

## Abstract

Background: This study aimed to investigate how myopia affects early event-related potential (ERP) components and neural oscillatory activity during a face inversion task in adolescents, in order to clarify the potential mechanisms by which degraded visual input influences facial structural encoding and neural dynamic regulation. Methods: Forty-eight adolescents aged 13–17 years participated, including 24 with myopia and 24 with normal vision. EEG signals were recorded while participants performed a classic upright–inverted face recognition task. A mixed-design ANOVA was applied to compare group differences in the amplitude and latency of the P1 and N1 components. Time–frequency analyses were conducted to assess task-related oscillatory power in the θ (4–7 Hz) and α (8–13 Hz) bands. Results: No significant group differences were found in P1 amplitude or latency, whereas the N1 latency was significantly longer in the myopia group (*p* = 0.049). Time–frequency analysis revealed significantly greater θ synchronization (*p* = 0.034) and greater α desynchronization (*p* = 0.018) within the early post-stimulus window corresponding to the P1 latency range in the myopia group, with a similar but nonsignificant trend observed within the window corresponding to the N1 range. The main effect of task confirmed a typical face inversion effect (FIE), characterized by smaller N1 amplitudes and longer latencies for inverted than upright faces (*p* < 0.05). Conclusions: Myopia in adolescents is associated with delayed neural processing during facial structural encoding and enhanced θ–α oscillatory activity within early post-stimulus periods, which may reflect altered early neural engagement in response to degraded visual input. These findings indicate that the effects of myopia extend beyond optical defocus to involve perceptual–cognitive integration, providing novel evidence for the neurodevelopmental characteristics and regulatory mechanisms of adolescent myopia.

## 1. Introduction

Face perception represents one of the most critical high-level visual functions supporting human social interaction, playing a central role in communication and social cognition [1]. The human brain exhibits remarkable specialization for facial processing, among which the face inversion effect (FIE) is one of the most prominent phenomena [2]. This effect refers to the markedly slower and less accurate recognition of inverted compared with upright faces, reflecting the disruption of holistic processing and a consequent reduction in the integration of identity and emotional information [3]. Empirical evidence indicates that face inversion interferes with the neural mechanisms underlying structural encoding, thereby impairing the extraction of configural relationships among facial features [4,5]. In addition, both social context and perceptual experience modulate the strength of the inversion effect. For instance, when faces are presented within socially meaningful contexts, the inversion effect is substantially attenuated, suggesting that face recognition involves not only structural encoding but also social–cognitive processes [6]. Further studies have shown that perceptual learning can alter the way individuals process inverted faces, highlighting the pivotal role of visual experience in shaping facial recognition ability [7].

In investigations of the temporal dynamics of face perception, event-related potential (ERP) techniques have been widely applied because of their high temporal resolution. Among ERP components, P1 and N1 (or N170) are considered to reflect the early perceptual and attentional stages of face processing [8]. The P1 component is associated with global perceptual processing and attentional allocation, representing an early stage of holistic visual integration, whereas the N1 component serves as a reliable neural index of facial recognition and categorization [9,10,11,12]. Previous studies have shown that upright faces elicit larger N170 amplitudes and shorter latencies than inverted faces, indicating that inversion disrupts the holistic processing mechanism [9,12]. Moreover, the emotional attributes of faces also modulate ERP responses; for instance, negative emotional faces (e.g., fearful expressions) often evoke enhanced P1 amplitudes, reflecting preferential attention to socially salient stimuli [13,14]. Collectively, these findings suggest that the face inversion effect reflects not only the specificity of visual structural encoding but also the intricate interplay between perceptual and social–cognitive processes at the neural level, with P1 and N1 components providing key electrophysiological evidence for understanding the temporal sequence and neural mechanisms of facial information processing.

In recent years, the incidence of adolescent myopia has risen sharply, becoming a major global public health concern [15]. Traditionally, myopia has been regarded primarily as an optical disorder of the refractive system; however, accumulating evidence indicates that prolonged exposure to blurred visual input can induce functional plasticity changes in the visual cortex [16,17]. Myopia not only reduces the quality of retinal imaging but also impairs top-down attentional control and spatial integration efficiency [18]. Empirical studies have reported that individuals with myopia perform worse than those with normal vision in tasks involving spatial attention, motion prediction, and figure–ground segregation, suggesting a constrained capacity for visuo-cognitive integration [19,20,21].

From a cognitive neuroscience perspective, visual experience not only shapes perceptual processing mechanisms but also profoundly influences higher-order cognitive processes such as attentional regulation and information encoding [22,23,24]. Prolonged near-work activity and exposure to reduced-quality visual input have been suggested to influence the engagement of large-scale visual processing pathways. Although the present study cannot determine the involvement of specific cortical regions, previous research indicates that such visual conditions may alter the efficiency of spatial and motion-related processing at early perceptual stages [25,26]. Impairments in dorsal stream function not only affect postural control and motor coordination but may also extend to early facial structural encoding circuits (occipitotemporal–parietal loop) [27]. Adolescence represents a critical period of heightened neurodevelopment and plasticity, during which sustained visual degradation may be associated with changes in how the visual cortical network is functionally engaged.

However, evidence regarding the impact of myopia on the neural processing of the face inversion task remains limited, particularly among adolescents. The face inversion paradigm not only reliably activates face-specific neural pathways [28] but also serves as an effective tool for probing how alterations in visual experience influence higher-order visual cognition [29]. Therefore, comparing the electrophysiological responses of adolescents with myopia and those with normal vision during this task may provide novel insights into the neurocognitive mechanisms underlying visual–cognitive development in myopia.

Based on this framework, the present study employed event-related potential (ERP) techniques combined with time–frequency analysis to compare EEG responses between myopic and normally sighted adolescents during the face inversion task. We analyzed both the amplitude and latency of the P1 and N1 components, as well as the oscillatory power of the θ (4–7 Hz) and α (8–13 Hz) frequency bands.

We hypothesized that, compared with the normal-vision group, the myopia group would exhibit prolonged N1 latency, greater θ synchronization, and greater α desynchronization during early perceptual processing, which may reflect altered early-stage neural engagement associated with degraded visual input.

## 2. Materials and Methods

### 2.1. Participants

A total of 48 male adolescents aged 13–17 years participated in the study, including 24 in the myopia group and 24 in the normal-vision group. The inclusion of male participants was based on methodological considerations related to EEG data acquisition. Because this study used a Ag/AgCl electrode cap, stable and consistent scalp–electrode contact is essential for maintaining low impedance and high-quality EEG signals. Hair thickness, density, and hairstyle can introduce variability in electrode contact; therefore, to ensure reliable and consistent data acquisition, we recruited participants with short and uniform hairstyles that facilitate EEG recording. In our accessible participant pool, these criteria were primarily met by male adolescents, and thus all participants were male. This rationale has also been acknowledged in the limitations paragraph of the Conclusions section (Section 5).

All participants were right-handed, had no history of neurological or psychiatric disorders, and completed standardized visual acuity and refractive examinations prior to the experiment. The degree of myopia ranged from −1.00 D to −5.00 D, measured by a certified optometrist using an auto-refractometer (Topcon KR-800, Topcon Corporation, Tokyo, Japan). In the normal-vision group, the spherical equivalent refractive error was within ±0.50 D. All participants had best-corrected visual acuity (BCVA) ≥ 1.0 (LogMAR ≤ 0). Basic demographic and ocular characteristics of the two groups are summarized in Table 1.

To ensure consistent visual input, all participants performed the experimental task while wearing their habitual optical correction. In the myopia group, all individuals used single-vision spectacles rather than contact lenses, minimizing potential differences in retinal image magnification or optical aberrations. The viewing distance was fixed at 70 cm for all participants.

Inclusion criteria were as follows: (1) age between 13 and 17 years; (2) refractive error between −1.00 D and −5.00 D for the myopia group, and within ±0.50 D for the normal-vision group; (3) BCVA ≥ 1.0 after correction; and (4) right-handedness. Exclusion criteria included: (1) other ocular abnormalities (e.g., astigmatism > 1.50 D, strabismus, or amblyopia); (2) a history of neurological or psychiatric illness; (3) use of medications that may affect visual or cognitive function; (4) history of brain injury or cranial surgery; and (5) fundus pathology or prior refractive surgery.

Although the refractive range (−1.00 to −5.00 D) covers both mild and moderate myopia, the sample size within each severity level was insufficient to support reliable subgroup analyses. Therefore, myopia severity was not further stratified, and all individuals with myopia were treated as a single group to avoid underpowered comparisons.

All participants and their legal guardians provided written informed consent prior to the experiment. The study protocol was approved by the Ethics Committee of the School of Physical Education, Ningxia Normal University (approval number: 20241201) and conducted in accordance with the Declaration of Helsinki.

### 2.2. Stimuli

Neutral-expression facial photographs were selected from a standardized face database (i.e., the Ekman and Friesen Pictures of Facial Affect [30]. All images were preprocessed by the database to remove external cues such as hair, ears, and jawline contours, resulting in gender-neutral facial stimuli without identifiable male–female characteristics. Therefore, no gender imbalance was present in the stimulus set, and face-gender effects could not influence the participants’ behavioral or neural responses.

All images were grayscale facial photographs standardized to a size of 300 × 400 pixels. At a viewing distance of 70 cm, the stimuli subtended a visual angle of approximately 6.4° (width) × 8.6° (height). Each face was presented in two orientations—upright and inverted—and the order of presentation was fully randomized to minimize sequence effects. The experiment employed a classic face inversion paradigm. Participants were seated approximately 70 cm from the monitor in a dimly lit, sound-attenuated room to ensure optimal viewing conditions and reduce environmental distractions.

Each trial began with a central fixation cross (“+”) presented for 500 ms, followed by a 300 ms display of either an upright or an inverted face. After stimulus offset, a blank screen was shown for a fixed 700 ms, during which participants were required to indicate the orientation of the face (upright or inverted) by pressing the corresponding key on a response pad. The next trial commenced immediately after the 700 ms interval, resulting in a constant inter-trial interval across the task.

Each participant completed four experimental blocks, with 60 trials per block (30 upright and 30 inverted faces), yielding a total of 240 trials. To prevent fatigue, a fixed 2 min break was provided between the four blocks, and participants resumed the task after the break.

### 2.3. EEG Recording and Analysis

EEG data were recorded using a NeuroScan acquisition system equipped with a 32-channel Ag/AgCl electrode cap arranged according to the international 10–20 system. The ground electrode was positioned at the midline frontal site (FPz), following standard NeuroScan procedures. The sampling rate was set to 1000 Hz to ensure sufficient temporal resolution for accurately capturing event-related potentials (ERPs) and their time–frequency dynamics. The reference electrode was initially placed at the left mastoid (A1) and was re-referenced offline to the averaged bilateral mastoids (A1 + A2) to minimize lateral reference bias. All electrode impedances were maintained below 10 kΩ. Synchronized event markers were recorded to indicate stimulus onset for precise temporal alignment of ERP and time–frequency analyses. Vertical and horizontal electro-oculogram (EOG) signals were simultaneously recorded to assist with artifact identification and removal.

Offline preprocessing was performed using Curry software and the EEGLAB toolbox (version 2021.1) running in MATLAB R2020b (MathWorks, Natick, MA, USA). Raw EEG signals were band-pass filtered (0.1–30 Hz) to remove DC drift and high-frequency muscle artifacts, and a 50 Hz notch filter was applied to suppress line noise. Artifact rejection was conducted using independent component analysis (ICA) to identify and remove components related to eye blinks, eye movements, and other physiological noise, followed by manual inspection. The cleaned EEG data were then segmented (epoched) from −200 ms to 800 ms relative to stimulus onset, including a 200 ms pre-stimulus baseline. Each epoch was baseline-corrected using the mean voltage of the pre-stimulus interval (−200 to 0 ms).

ERP analyses focused on the early sensory components P1 and N1, defined as a positive deflection occurring approximately 80–130 ms after stimulus onset (P1) and a negative deflection at 150–200 ms (N1). Based on previous literature and the characteristics of the task, analyses were restricted to five occipital–parietal electrodes (O1, Oz, O2, POz, Pz), which are most sensitive to early visual responses.

For each participant and each condition, ERP waveforms were first averaged across all valid trials at each of the five electrodes. The mean or peak amplitudes within the P1 and N1 time windows were then extracted separately from each electrode. These values were subsequently averaged across the five occipital–parietal electrodes to obtain a single representative P1 and N1 amplitude and latency measure per participant for each condition. This procedure ensured that the ERP indices reflected activity consistently across the predefined occipital–parietal region of interest and provided the spatial–temporal references for subsequent time–frequency (ERSP) analyses.

To examine dynamic changes in θ (4–7 Hz) and α (8–13 Hz) oscillatory activity during the task, time–frequency decomposition was performed using a short-time Fourier transform (STFT) implemented with custom MATLAB scripts rather than EEGLAB’s default wavelet-based functions. Preprocessed EEG signals were segmented from −200 to 800 ms relative to stimulus onset. A 500 ms Hanning window with a 50 ms step size was applied to each epoch, providing stable spectral estimates for low-frequency θ (4–7 Hz) and α (8–13 Hz) rhythms by capturing multiple oscillatory cycles, particularly near the lower bound of the θ band. For each time–frequency point, spectral power was computed as the squared magnitude of the complex STFT output. We acknowledge that the use of a relatively long analysis window reduces temporal precision and introduces temporal smearing across adjacent time points. Therefore, the resulting time–frequency representations are interpreted as reflecting broader early post-stimulus periods corresponding to the P1 and N1 latency ranges, rather than isolating neural activity strictly confined to the peak latencies of these ERP components. Baseline normalization (−200 to 0 ms) was then applied to produce ERSP indices for subsequent statistical analyses.

The STFT was applied to each windowed signal segment x(t), and the time-frequency representation was computed as follows:

(1) $$\mathrm{STFT}(t,f)\;=\;\int_{-\infty }^{\infty }\;x(\tau )\cdot w(\tau -t)\cdot e^{-j2\pi f\tau }d\tau$$

Here, x(τ) represents the input EEG signal; w(τ−t) denotes the Hanning window centered at time; f is the frequency of interest; and t indicates the time point relative to stimulus onset. The output of the STFT(t,f) is a complex-valued function that contains both amplitude and phase information, which can be used to calculate time-resolved spectral power across specific frequency bands.

To obtain the temporal evolution of spectral power across frequencies, the power spectrum at each time-frequency point was computed as:

(2) $$P(t,f)\;=\;{\left|\mathrm{STFT}(t,f)\right|}^{2}$$

To enable across-trial comparisons and statistical analysis, the power spectra were normalized to yield relative power change (RPC) values. The 200 ms pre-stimulus interval (−200 to 0 ms) served as the baseline. For each frequency fff and time point ttt, RPC was calculated as:

(3) $$\mathrm{RPC}(t,f)\;=\frac{P(t,f)-\bar{P_{\mathrm{baseline}\;}}\left(f\right)}{\bar{P_{\mathrm{baseline}\;}}\left(f\right)}$$

where $\bar{P_{\mathrm{baseline}\;}}\left(f\right)$ is the mean power at frequency f during the baseline window, and RPC(t,f) represents the relative change in power at time t and frequency f. Positive RPC values indicate post-stimulus power increases relative to baseline, whereas negative values denote power decreases.

To focus on activity in the theta and alpha frequency bands, power values were averaged within the 4–7 Hz and 8–13 Hz ranges, respectively:

(4) $$\mathrm{RP}C_{\theta }\left(t\right)\;=\;\frac{1}{N_{\theta }}\sum_{f=4}^{7}\;\mathrm{RPC}(t,f),\;\mathrm{RP}C_{\alpha }\left(t\right)\;=\frac{1}{N_{\alpha }}\sum_{f=8}^{13}\;\mathrm{RPC}(t,f)$$

Here $N_{\theta }$ and $N_{\alpha }$ denote the number of frequency points within the respective bands. The final analysis focused on the 0–500 ms post-stimulus time window, during which the relative power change (RPC) values were extracted. The spatial region of interest was constrained to the same set of electrodes used in the ERP analysis, enabling cross-method comparisons and potential data integration. For statistical analysis, the RPC values within the 0–500 ms window were averaged across time for each participant, yielding condition-wise mean power values. These values were then used for within- and between-group statistical comparisons.

### 2.4. Statistical Analysis

All statistical analyses were performed using SPSS version 26.0 (IBM Corp., Armonk, NY, USA), with the level of significance set at *p* < 0.05. To examine the effects of experimental conditions on neural responses, separate 2 × 2 mixed-design analyses of variance (ANOVAs) were conducted for the ERP components (P1 and N1) and for the relative power changes in the θ (4–7 Hz) and α (8–13 Hz) frequency bands. The design included one between-subject factor, Group (myopia vs. normal vision), and one within-subject factor, Task (upright vs. inverted faces). The analysis window was defined as 0–500 ms following stimulus onset.

Prior to the ANOVA, data normality was assessed using the Shapiro–Wilk test, and the Mauchly’s test of sphericity was applied to evaluate the assumption of homogeneity for within-subject effects. When sphericity was violated, Greenhouse–Geisser corrections were applied to adjust the degrees of freedom. In cases where significant interaction effects were observed, simple effects analyses were performed to explore group differences under each task condition or within-group variations across tasks.

To reduce interindividual variability, all ERP amplitudes were baseline-corrected using the −200 to 0 ms pre-stimulus interval, and time–frequency data were normalized relative to the same baseline period, ensuring comparability across conditions. When multiple comparisons were required, Bonferroni corrections were applied to control the Type I error rate. For variables that violated normality assumptions or contained extreme outliers, nonparametric tests (e.g., Mann–Whitney U test) were used for confirmatory analyses.

## 3. Results

### 3.1. ERP Results

**Figure 1:**
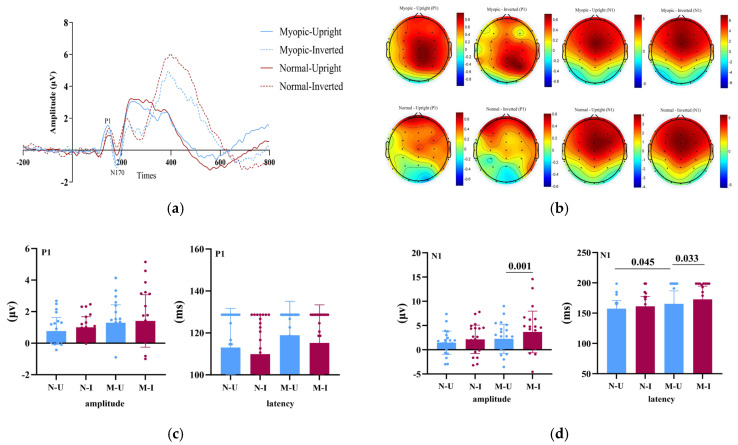
Group-level ERP results during the face inversion task. (**a**) Grand-averaged ERP waveforms for the myopia and normal-vision groups under upright and inverted face conditions. Shaded areas indicate the P1 (80–130 ms) and N1 (150–200 ms) time windows. (**b**) Scalp topographies of the P1 and N1 components for both groups under different task conditions, averaged across the corresponding time windows and regions of interest (ROIs). (**c**) Bar plots showing group comparisons of P1 amplitude and latency. N-U = Normal–Upright; N-I = Normal–Inverted; M-U = Myopic–Upright; M-I = Myopic–Inverted. (**d**) Bar plots showing group comparisons of N1 amplitude and latency. N-U = Normal–Upright; N-I = Normal–Inverted; M-U = Myopic–Upright; M-I = Myopic–Inverted. Statistical analyses were conducted only on predefined occipital–parietal electrodes (O1, Oz, O2, POz, Pz) rather than on all scalp electrodes.

Figure 1 summarizes the grand-average ERP findings. Specifically, Figure 1a shows group-averaged waveforms across task conditions, highlighting the temporal dynamics of the P1 and N1 components; Figure 1b depicts their scalp topographies; Figure 1c,d report the statistical comparisons for peak amplitude and latency.

For the P1 amplitude, neither the main effect of Group, F(1, 46) = 2.40, *p* = 0.128, ηp^2^ = 0.050, nor the main effect of Task, F(1, 46) = 1.57, *p* = 0.216, ηp^2^ = 0.033, reached significance. The interaction was also nonsignificant, F(1, 46) = 0.17, *p* = 0.679, ηp^2^ = 0.004.

For the P1 latency, there were no significant effects of Group, F(1, 46) = 1.28, *p* = 0.246, ηp^2^ = 0.027, Task, F(1, 46) = 2.83, *p* = 0.099, ηp^2^ = 0.058, or their interaction, F(1, 46) = 0.02, *p* = 0.903, ηp^2^ = 0.000.

For the N1 amplitude, a 2 (Group: myopia vs. normal vision) × 2 (Task: upright vs. inverted faces) mixed ANOVA revealed no main effect of Group, F(1, 46) = 1.76, *p* = 0.191, ηp^2^ = 0.037, but a significant main effect of Task, F(1, 46) = 12.91, *p* = 0.001, ηp^2^ = 0.219, with inverted faces eliciting larger (more negative) N1 amplitudes than upright faces (Figure 1c). The Group × Task interaction was not significant, F(1, 46) = 1.51, *p* = 0.225, ηp^2^ = 0.032, indicating comparable task-related modulation across groups.

For the N1 latency, the mixed ANOVA showed a significant main effect of Group, F(1, 46) = 4.82, *p* = 0.049, ηp^2^ = 0.095, with longer N1 latencies in the myopia group relative to controls. The main effect of Task was also significant, F(1, 46) = 5.53, *p* = 0.023, ηp^2^ = 0.107; latencies were longer for inverted than upright faces. The interaction was not significant, F(1, 46) = 0.58, *p* = 0.451, ηp^2^ = 0.012.

### 3.2. ERSP Results

**Figure 2:**
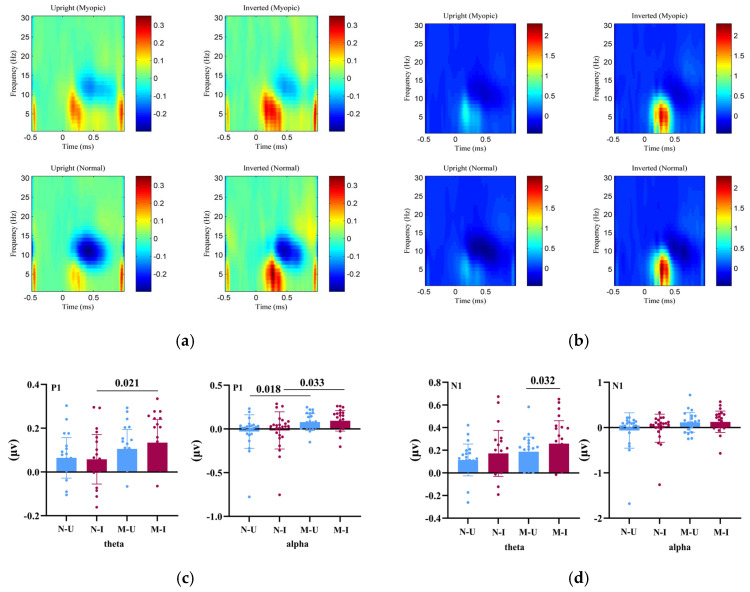
Event-related spectral perturbation (ERSP) results for θ and α oscillatory activity during early visual processing. (**a**) Time–frequency representations of baseline-normalized θ (4–7 Hz) and α (8–13 Hz) power changes within the early post-stimulus window corresponding to the P1 latency range across groups and task conditions. (**b**) ERSP maps within the subsequent early window corresponding to the N170 (N1) latency range, illustrating dynamic patterns of θ synchronization and α desynchronization associated with early stages of face processing. (**c**) Bar plots showing statistical comparisons of θ synchronization and α desynchronization for the P1-related early window. (**d**) Bar plots showing statistical comparisons of θ synchronization and α desynchronization for the N1-related early window. N-U = Normal–Upright; N-I = Normal–Inverted; M-U = Myopic–Upright; M-I = Myopic–Inverted.

Figure 2 illustrates the overall findings from the time–frequency analysis. Because the STFT used a 500 ms analysis window, the oscillatory results reflect broader early post-stimulus periods corresponding to the P1 (≈80–130 ms) and N1 (≈150–200 ms) latency ranges. Figure 2a shows the event-related spectral perturbation (ERSP) maps for θ (4–7 Hz) and α (8–13 Hz) activity within the early time window corresponding to the P1 latency range. Figure 2b displays ERSP maps for the subsequent early window corresponding to the N1 latency range. Figure 2c,d summarize statistical comparisons for θ and α synchronization/desynchronization across these early windows.

For the P1 θ band, a 2 (Group: myopia vs. normal vision) × 2 (Task: upright vs. inverted faces) mixed ANOVA revealed a significant main effect of Group, F(1, 46) = 4.75, *p* = 0.034, ηp^2^ = 0.094, indicating greater θ synchronization (i.e., a larger θ-band power increase relative to baseline) in the myopia group compared with controls.

The main effect of Task was not significant, F(1, 46) = 0.95, *p* = 0.334, ηp^2^ = 0.020, nor was the Group × Task interaction, F(1, 46) = 2.41, *p* = 0.127, ηp^2^ = 0.050, suggesting comparable task-related modulation of θ activity across groups.

For the P1 α band, there was also a significant main effect of Group, F(1, 46) = 6.04, *p* = 0.018, ηp^2^ = 0.116, reflecting greater α desynchronization (i.e., a stronger α-band power decrease relative to baseline) in the myopia group compared with controls. No significant Task effect, F(1, 46) = 0.83, *p* = 0.366, ηp^2^ = 0.018, or Group × Task interaction, F(1, 46) = 0.00, *p* = 0.961, ηp^2^ ≈ 0.000, was found.

For the N1 θ band, the main effect of Group did not reach significance, F(1, 46) = 3.26, *p* = 0.078, ηp^2^ = 0.066. However, there was a significant main effect of Task, F(1, 46) = 7.94, *p* = 0.007, ηp^2^ = 0.147, indicating greater θ synchronization (i.e., a larger θ-band power increase relative to baseline) for inverted faces than for upright faces. The interaction was not significant, F(1, 46) = 0.10, *p* = 0.756, ηp^2^ = 0.002.

For the N1 α band, neither the Group, F(1, 46) = 3.72, *p* = 0.060, ηp^2^ = 0.075, nor the Task, F(1, 46) = 0.98, *p* = 0.166, ηp^2^ = 0.041, showed significant effects, and the interaction was also nonsignificant, F(1, 46) = 0.66, *p* = 0.419, ηp^2^ = 0.014.

This pattern indicates that both groups exhibited similar θ–α power modulation across task conditions.

## 4. Discussion

The present study systematically compared the neural activity characteristics of adolescents with myopia and those with normal vision during the early stages of visual processing using a face inversion task. By integrating ERP and ERSP analyses, the study revealed how myopia influences the perceptual–cognitive coupling mechanisms underlying facial recognition. Specifically, we examined the amplitude, latency, and associated oscillatory activity of the P1 and N1 components. The results showed no significant group differences in either the amplitude or latency of the P1 component. The P1 is primarily generated in the primary and secondary visual cortices (V1/V2) of the occipital lobe and reflects rapid sensory responses to low-level visual features such as luminance and contrast [31], which can be further modulated by attentional processes [32]. Therefore, these findings suggest that myopia does not substantially alter cortical responses during the initial visual input stage, indicating that although optical defocus leads to blurred retinal imaging, the bottom-up signal transmission along the retina–V1/V2 pathway remains largely preserved. In other words, the neural differences associated with myopia are more likely to emerge during facial structural encoding and integrative processing, rather than at the level of primary perceptual input [33]. This interpretation aligns with the functional localization of the P1 component, implying that the neural alterations in myopia primarily involve higher-order cognitive integration rather than early visual input processing [9].

In this study, adolescents with myopia exhibited a significant delay in N1 latency, which may indicate altered neural processing efficiency during early facial structural encoding. Although prior source-localization studies have linked the N1 component to occipitotemporal activity [9,34], our EEG configuration does not allow identification of specific cortical generators. Therefore, the observed delay should be interpreted as reflecting differences in early-stage visual processing rather than changes within any particular region.

Previous research suggests that prolonged exposure to visually degraded input may influence the overall engagement of both feedforward and feedback processes, potentially increasing the reliance on attentional or working-memory mechanisms to maintain perceptual performance [35]. While the present study cannot specify the pathways involved, the pattern of delayed responses may be consistent with broader alterations in the coordination of early perceptual processes under reduced visual clarity.

From the perspective of large-scale visual networks, sustained low-quality visual signals have been associated in prior literature with changes in the efficiency of bottom-up structural cue extraction [25,26,27]. Although our data cannot verify involvement of specific pathways, such literature-informed interpretations provide a useful framework for understanding how degraded input might shape the temporal characteristics of early visual encoding.

At the same time, feedback modulation from parietal or prefrontal cortices may become less effective, contributing to less synchronized early processing across the face-processing network [36]. Such changes in early feedforward feedback coordination could manifest temporally as delayed N1 responses, reflecting group-associated differences in early visual information processing rather than definitive effects of neural plasticity [17].

Although the group difference in N1 amplitude did not reach significance, we observed a robust main effect of task: inverted faces elicited smaller (more negative) N1 amplitudes than upright faces, consistent with the classic face inversion effect (FIE). This pattern aligns with the well-established view that inversion disrupts holistic and configural processing, leading to increased reliance on feature-based analysis [37]. Prior source-localization studies have reported that face inversion alters the balance between global-configural and part-based processing pathways [38,39,40]; however, our EEG montage does not allow identification of the specific cortical regions involved. The present findings are therefore best interpreted as reflecting the characteristic perceptual shift associated with face inversion rather than the activity of particular neural sources.

In the present study, the absence of a significant Group × Task interaction suggests that although individuals with myopia exhibited delayed neural processing in temporal dynamics, their face perception still followed the typical dual-route architecture of holistic and local processing, without a restructuring of face-specific neural mechanisms [41]. This finding implies that reduced visual input quality primarily affects the speed and efficiency of neural resource allocation, rather than altering the hierarchical organization of processing strategies [42]. Individuals with myopia may therefore recruit additional neural resources to maintain perceptual stability, sustaining normal holistic processing at the expense of increased cognitive and energetic load [16,43].

At the level of neural oscillations, the present study found that adolescents with myopia exhibited significantly greater θ synchronization and greater α desynchronization during the P1 stage compared with those with normal vision, reflecting stronger baseline-normalized oscillatory modulations during early visual processing. This enhancement may result from the increased perceptual integration load caused by long-term degradation in visual input quality. When visual signals are blurred or contrast is reduced, the primary visual cortex (V1/V2) must engage a broader population of synchronized neurons to enhance signal coherence and counteract noise interference during the encoding of low–spatial-frequency information [44,45].

Theta oscillations are closely associated with attentional allocation and perceptual integration, and their enhancement is considered a coordinative mechanism that helps the neural system maintain input stability under high-load or uncertain conditions [46]. In contrast, alpha oscillations are closely related to selective attention and inhibitory control. Rather than reflecting increased absolute power, stronger α desynchronization (i.e., a greater reduction in α-band power relative to baseline) is widely interpreted as enhanced engagement of attentional and inhibitory mechanisms, enabling the visual system to suppress irrelevant information and reduce external noise when sensory input is degraded [47,48].

During the P1 period, adolescents with myopia showed greater θ–α–band changes relative to baseline, which may reflect enhanced engagement of early oscillatory processes in response to degraded sensory input. Rather than indicating a compensatory mechanism, this pattern may suggest that the visual system allocates additional early-stage neural resources to stabilize perceptual processing when the incoming signal is less precise [49]. Such enhanced synchronization/desynchronization dynamics could reflect modifications in how local and cross-frequency interactions are recruited during early perceptual analysis, without implying reorganization of cortical oscillatory networks [50].

Although the θ and α effects during the N1 window did not reach statistical significance, both bands showed a trend toward increased modulation. This pattern may indicate that these early oscillatory adjustments extend into the stage of structural encoding, consistent with previous evidence suggesting that θ–α interactions play a sustained role in maintaining processing stability across perceptual stages [50,51]. Accordingly, adolescents with myopia may engage in oscillatory activity more strongly across multiple stages of face processing to support structural integration under reduced visual clarity.

In addition, the main effect of task revealed that inverted faces elicited stronger θ-band modulation than upright faces, consistent with the increased processing demands associated with face inversion. Disrupting holistic/configural information forces the system to adopt a more feature-based analytical strategy, thereby increasing the reliance on attentional and perceptual resource allocation [2]. Under such conditions, the heightened θ-band activity may reflect stronger large-scale coordination between posterior perceptual regions and higher-order attentional networks [52,53,54].

Theta oscillations are widely considered a signature of cognitive control and cross-regional coordination. Their enhancement under greater task demands suggests that when perceptual complexity increases, the neural system supports coherent feature binding by strengthening network-level synchronization [55,56]. The greater θ-band modulation observed in the myopia group may therefore reflect an increased reliance on higher-level control resources to maintain perceptual stability when structural information is degraded. This pattern is consistent with a processing-load account in which reduced visual precision and increased task complexity together lead to stronger engagement of oscillatory mechanisms [55].

In addition, several behavioral and lifestyle factors commonly associated with adolescent myopia—such as near-work duration, outdoor activity levels, and general cognitive ability—were not measured in the present study. Prior research indicates that prolonged near-work exposure and reduced outdoor time are linked to both myopic progression and alterations in visual behavior or ocular development. For instance, in a large survey of school-aged youth, longer near-work duration and lower frequency of outdoor activities were significantly associated with higher odds of myopia [57]. Longitudinal studies have similarly shown that increased time outdoors can decrease the risk or slow progression of myopia [58]. These lifestyle factors might, in theory, also affect attentional control, visual processing load, or neural oscillatory dynamics, thereby representing potential confounds in our EEG findings.

Therefore, the absence of direct measures of near-work, outdoor time, cognitive ability in our study remains a limitation. Future studies should systematically assess these variables to disentangle their contributions to neural differences in myopia.

## 5. Conclusions

Using a face inversion paradigm, this study compared electrophysiological characteristics of adolescents with myopia and those with normal vision during the early stages of visual processing. By integrating ERP and time–frequency analyses, we examined how myopic visual input is associated with differences in facial structural encoding and early oscillatory activity. The results revealed that adolescents with myopia showed a significant delay in N1 latency, which may reflect altered neural processing efficiency during structural encoding. In addition, they exhibited greater θ synchronization and greater α desynchronization during the early post-stimulus period corresponding to the P1 window, with a similar but nonsignificant trend during the N1 window. These patterns may indicate enhanced early-stage neural engagement when processing visual information of reduced precision.

Overall, our findings suggest that myopia may be associated with differences in early perceptual–cognitive processing, reflected in delayed temporal responses and stronger modulation of θ–α oscillatory activity. Although both groups showed a typical face inversion effect with similar processing patterns, the myopia group demonstrated stronger oscillatory modulation and slower structural encoding, which may reflect adjustments in early neural resource allocation rather than definitive compensatory mechanisms. These interpretations should be considered preliminary, and future longitudinal or mechanistic studies are needed to clarify the functional significance of these early neural differences.

This study revealed the spatiotemporal characteristics of early visual processing in adolescents with myopia during a face inversion task from a neurophysiological perspective. The findings confirmed an association between myopic status and enhanced neural oscillatory activity, providing new evidence for how visual experience shapes the facial processing network. Nevertheless, several limitations should be acknowledged.

(1) The sample size and demographic characteristics may limit the generalizability of the findings. All participants in this study were healthy male adolescents, which restricts the ability to generalize the findings to female adolescents or to broader age groups. Although male participants were selected based on methodological considerations related to EEG acquisition (i.e., the need to ensure stable scalp–electrode contact when using Ag/AgCl electrode caps), future studies should include more gender-diverse samples to examine potential sex-related differences in neural processing. In addition, although the refractive range (−1.00 to −5.00 D) encompassed both mild and moderate myopia, the sample size within each severity category was insufficient to support reliable stratified analyses. Moreover, dose–response relationships between spherical equivalent (SE) or axial length and ERP/oscillatory indices were not assessed due to limited statistical power. Future research with larger, stratified samples is needed to clarify how myopia severity, onset age, and refractive progression rate modulate neural signatures of visual processing.

(2) EEG data in this study were collected using a 32-channel Ag/AgCl electrode system. Although this configuration is sufficient for capturing robust ERP signals from posterior visual regions, its spatial resolution is limited and does not allow high-precision cortical topography or detailed assessment of broader cortical network involvement. Consequently, our analyses focused on occipital–parietal electrodes, which are most sensitive to early visual components such as P1 and N1. Future research using high-density EEG or source reconstruction techniques (e.g., sLORETA, dSPM) is needed to more precisely evaluate distributed cortical contributions to face processing.

(3) Moreover, the study did not collect objective measures of near-work exposure, outdoor activity time, or general cognitive ability. These variables may influence both myopia development and oscillatory patterns related to attention and perceptual processing. Future work should include standardized assessments of visual behavior and cognitive functioning to better dissociate their contributions to early neural processing differences in myopic adolescents.

(4) Adolescence represents a critical period of heightened neuroplasticity. Understanding how myopia influences the development of neural networks may help clarify the principles of visual–cognitive maturation and inform targeted intervention strategies. Future studies could explore how different forms of motor-based or visuo-cognitive interventions, such as visual–motor training or cognitive–motor integration tasks, enhance neuroplasticity and cognitive efficiency in individuals with myopia.

## Figures and Tables

**Table 1:** Basic Information of Participants.

Variable	Normal (n = 24)	Myopia (n = 24)
Age (years)	15.1 ± 1.5	15.2 ± 1.1
BMI	20.8 ± 2.3	21.1 ± 2.5
Refractive Error (D)	−0.5~0.5	−2.87 ± 1.21 **
Axial length (mm)	23.7 ± 0.8	25.3 ± 1.0 *
Dominant Hand	Right	Right

Note: vs. Normal, * *p <* 0.05, ** *p <* 0.01; Body Mass Index (BMI) was calculated as weight in kilograms divided by the square of height in meters (kg/m^2^); Data are presented as mean ± standard deviation (SD).

## Data Availability

The experimental data generated in this study are not publicly available due to privacy protection and ethical restrictions involving adolescent participants. The data were provided by Chen Peng and may be made available from the corresponding author upon reasonable request and with permission from the Ethics Committee of the School of Physical Education, Ningxia Normal University.
